# Dendritic Cell Editing by Activated Natural Killer Cells Results in a More Protective Cancer-Specific Immune Response

**DOI:** 10.1371/journal.pone.0039170

**Published:** 2012-06-19

**Authors:** Barbara Morandi, Lorenzo Mortara, Laura Chiossone, Roberto S. Accolla, Maria Cristina Mingari, Lorenzo Moretta, Alessandro Moretta, Guido Ferlazzo

**Affiliations:** 1 Department of Experimental Medicine, University of Genoa, Genoa, Italy; 2 Department of Biotechnology and Life Sciences, University of Insubria, Varese, Italy; 3 Istituto G.Gaslini, Genoa, Italy; 4 Department of Surgical and Morphological Sciences, University of Insubria, Varese, Italy; 5 Istituto Di Ricovero e Cura a Carattere Scientifico A.O.U. San Martino-IST-Istituto Nazionale per la Ricerca sul Cancro, Genoa, Italy; 6 Department of Human Pathology, University of Messina, Messina, Italy; University of Palermo, Italy

## Abstract

Over the last decade, several studies have extensively reported that activated natural killer (NK) cells can kill autologous immature dendritic cells (DCs) in vitro, whereas they spare fully activated DCs. This led to the proposal that activated NK cells might select a more immunogenic subset of DCs during a protective immune response. However, there is no demonstration that autologous DC killing by NK cells is an event occurring *in vivo* and, consequently, the functional relevance of this killing remains elusive. Here we report that a significant decrease of CD11c^+^ DCs was observed in draining lymph nodes of mice inoculated with MHC-devoid cells as NK cell targets able to induce NK cell activation. This *in vivo* DC editing by NK cells was perforin-dependent and it was functionally relevant, since residual lymph node DCs displayed an improved capability to induce T cell proliferation. In addition, in a model of anti-cancer vaccination, the administration of MHC-devoid cells together with tumor cells increased the number of tumor-specific CTLs and resulted in a significant increase in survival of mice upon challenge with a lethal dose of tumor cells. Depletion of NK cells or the use of perforin knockout mice strongly decreased the tumor-specific CTL expansion and its protective role against tumor cell challenge. As a whole, our data support the hypothesis that NK cell-mediated DC killing takes place *in vivo* and is able to promote expansion of cancer-specific CTLs. Our results also indicate that cancer vaccines could be improved by strategies aimed at activating NK cells.

## Introduction

Natural Killer (NK) cells, which were originally identified as lymphoid cells capable of lysing a number of tumor cell lines in the absence of previous stimulation *in vivo* or *in vitro* [1]–[3], are now appreciated as multifunctional innate immune cells [4]–[6]. Their activation is guided by a balance of signals given by different groups of activating [7] and inhibitory receptors [8]–[11], the latter recognizing MHC class I molecules on target cells.

Recently, the key role for a cooperative dialogue between DCs and natural killer (NK) cells in triggering the immune response has emerged [12]–[17]. It has been shown that their interaction results in a bi-directional activation and in the development of a Th1 and CTL mediated response [18]–[21]. In humans, at least *in vitro*, this cross-talk also results in the lysis of immature DCs, while mature DCs are protected [13], [15]. The activating receptor NKp30 and DNAM-1 are critical receptors for DC lysis, while resistance to lysis is mediated by up-regulation of MHC class I molecules during DC maturation [15], [22]–[24].

On the basis of these in vitro findings, it has been proposed that the killing of immature DCs by NK cells should promote the survival of the most immunogenic DCs (i.e. mature DCs), favoring initiation of an efficient and protective immune response [25]–[27].

*In vivo*, DC/NK-cell interactions might occur in lymphoid organs as well as in non lymphoid tissues [28]. Adoptively transferred, *ex vivo* generated, immature DCs have been reported to be rapidly eliminated by NK cells via TNF-related apoptosis-inducing ligand (TRAIL) [29], [30]. Similarly, it has been shown that the transplantation of alloreactive NK cells can suppress T-cell-mediated graft-versus-host disease by eliminating host DCs [31], [32]. During chronic viral infections, an aberrant DC susceptibility to NK cell-mediated lysis resulted in an accumulation of poorly immunogenic DCs in lymph nodes, causing progressive immune dysfunction [33]. On the other hand, DC lysis by NK cells could also negatively regulate the duration of virus-specific T cell responses *in vivo* by limiting exposure of T cells to infected antigen-presenting cells [34].

However, DC killing by autologous NK cells *in vivo* has not been directly demonstrated to date and the potential relevance of this lysis during a physiological immune response remains to be evaluated.

A number of *in vivo* models have provided evidence that NK cell recognition of MHC class I-deficient target cells results in an enhanced generation of CTLs against tumors [21], [35]. In these experimental models, activated NK cells produce cytokines that, in turn, appear to first promote DC activation and subsequently a protective CTL response against parental tumors. This prompted us to investigate whether, during a protective immune response against tumors, activated NK cells might also select a more immunogenic subset of DCs. We show here that DC editing occurs *in vivo* and that this phenomenon plays a key role for tumor-specific CTL development and mice survival in a murine model of tumor vaccination.

## Results and Discussion

### Activation of NK Cells in Peripheral Tissues Results in a Perforin-dependent Decrease of DC Content in Draining Lymph Nodes

**Figure 1:**
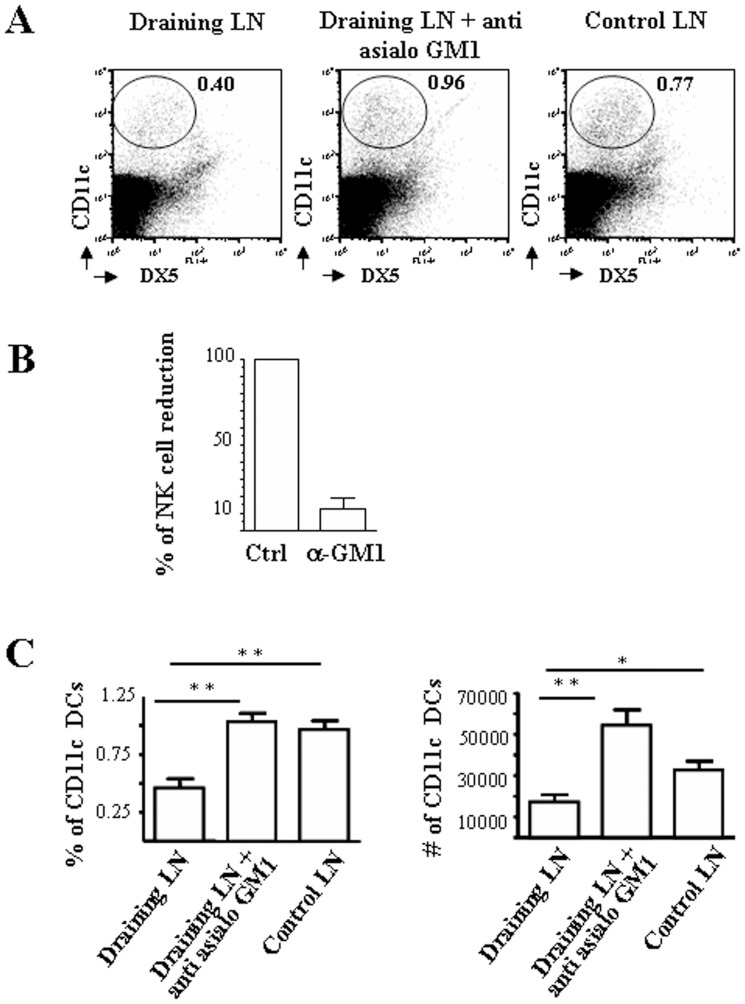
Subcutaneous administration of MHC-negative cells results in an NK cell-dependent decrease of CD11c^bright^ DCs in the draining lymph nodes. A: representative analyses of mononuclear cells isolated from either draining or controlateral (Control LN) lymph nodes of mice depleted or not of NK cells (Draining LN + anti-asialo GM1). B: NK cells are efficiently depleted in mice upon administration of anti-asialo GM1 mAbs. C: the percentage (left) and the absolute number (right) of DCs among mononuclear cells isolated from lymph nodes. Bars represents mean values and SD of five independent experiments (three mice per group). ** =  p < 0,001; * =  p < 0,005.

Mice were inoculated s.c. with YAC-1, a MHC-devoid cell line, as an NK cell target able to induce NK cell activation. After 36 h, DCs derived from both draining and controlateral LN were analyzed. As shown in figure 1, both the percentage and the absolute number of CD11c^+^ DCs were dramatically decreased in draining LNs compared to controlateral LNs (p = 0.0029 for the percentage and p = 0.007 for absolute number). *In vivo* depletion of NK cells by injecting anti-asialo-GM1 monoclonal antibodies (mAbs) reverted this phenomenon, confirming the major role played by NK cells. In draining LNs from NK cell-depleted mice, both the percentage and the absolute number of CD11c^+^ DCs were comparable with controlateral LNs, indicating that NK cells should be involved in the decrease of DCs. Anti-asialo GM1 mAb treatment led to a decrease of at least 80% of NK cells (Figure 1,B). These results suggested that the decline in DC number observed in draining LN was NK cell-dependent and apparently consequent to NK cell activation upon recognition of MHC-devoid cells.

One possible explanation for the observed reduction of DC cell numbers upon NK cell activation is the release of specific cytokines, by NK cells, able to influence the survival of DCs or their ability to migrate from the periphery to the LN. Alternatively, NK cells may affect the number of DCs in draining LN by direct lysis.

To elucidate the mechanism underlying the decline in DC numbers, we repeated the same experiment in perforin knockout (pfn^−/−^) mice, since perforin is an essential molecule for NK cell cytotoxicity. In the pfn^−/−^ mice, there were no differences in the number and percentage of CD11c^+^ DCs found in draining and controlateral LNs (not shown). These data suggest that perforin-dependent cytotoxicity might represent a pathway in the NK cell-dependent decrease of LN DCs.

### NK Cell Activation Selects Lymph Node DC with Higher T Cell Activating Capability

NK cells have been shown to kill immature DCs *in vitro* [13], [15]. One interpretation for the specific killing of healthy autologous cells by NK cells was that NK cells might act to control the quality of DCs undergoing maturation [26]. This hypothesis implies that NK cells would prevent the survival of less immunogenic immature DCs, which would induce inappropriate, low affinity T-cell priming, eventually resulting in a state of tolerization. The final outcome of this DC editing process mediated by NK cells might therefore be the selection of more immunogenic DCs, due to the removal of DCs that would fail to mediate optimal T cell priming.

**Figure 2:**
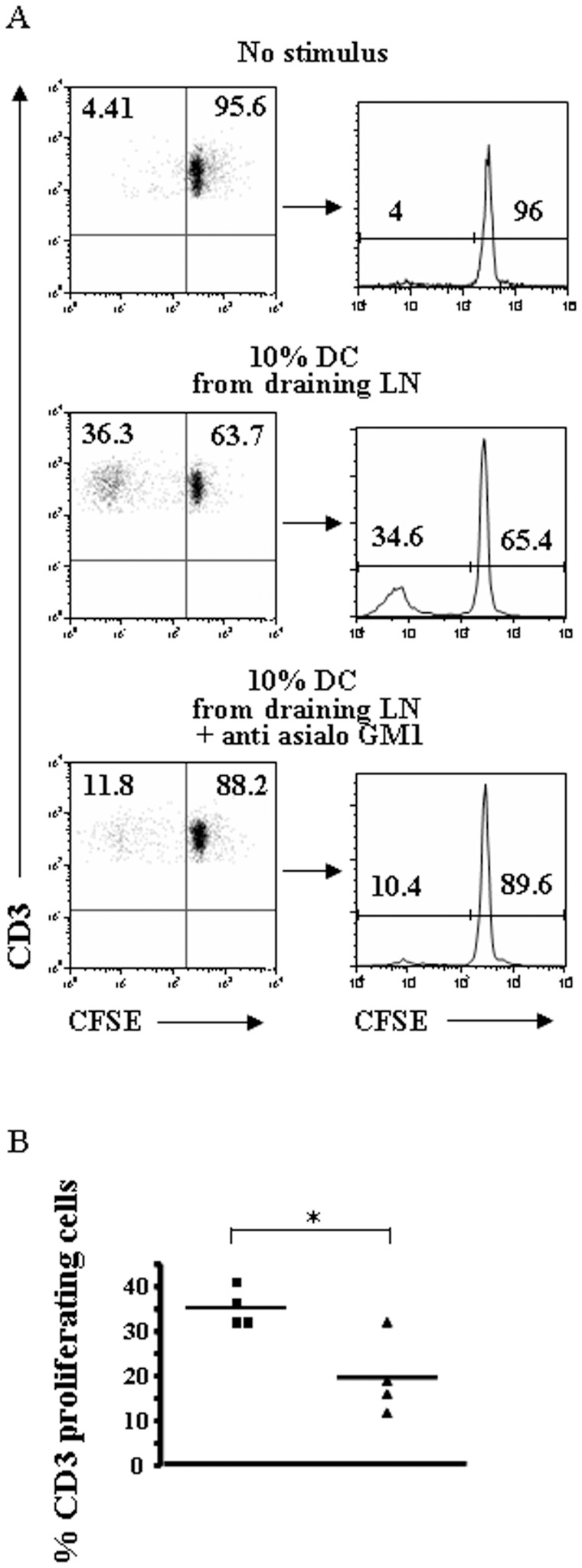
Peripheral activation of NK cells results in the selection of lymph node DCs with higher T cell activating capabilites. Draining lymph node DCs of mice injected subcutaneously with MHC^neg^cells were sorted and cultured in the presence of allogeneic splenocytes previously labeled with CFSE. A: Splenocytes were cultured alone (No stimulus) or with 10% of highly purified lymph node DCs from mice NK cell depleted (10% DC draining LN + anti-asialo GM1) or control (10% DC draining LN) before administration of MHC-negative target cells. CFSE dilution of the splenocytes at 6 days of culture is shown. NK cell depletion compromises the induction of proliferation by LN DCs. Data are representative of four independent experiments summarized in panel B: ▪ = 10% DC draining LN; ▴ = 10% DC draining LN + anti-asialo GM1. * =  p<0,02.

In order to verify whether this mechanism is functionally relevant in vivo, we tested whether DCs persistent in the draining LN after NK cell editing were phenotipically and functionally more immunogenic. Groups of mice that were either NK cell depleted or not were inoculated s.c. with YAC-1 cells. After 36 h DCs from the draining and controlateral lymph nodes were analyzed for the expression of co-stimulatory molecules and maturation markers such as CD40, CD80, CD83, CD86 by flow cytometry and for IL12p35, IL-12p40 and IL23p19 mRNA by real time PCR. In addition, we assessed whether DCs persistent in draining LN upon NK cell editing presented higher T cell activating capability. Splenocytes from C57BL/6 mice were stimulated with DCs sorted from lymph nodes of BALB/c mice injected with YAC-1 cells 24 h before lymph node removal. Six days later, the rate of proliferation was evaluated as loss of CFSE dye. Although significant differences for the analyzed surface molecules or cytokines were not discernable (not shown), allogeneic T cell proliferation was significantly decreased when DCs from NK cell-depleted mice were used as stimulus (Figure 2). These results indicate that NK cell-mediated decrease of DC number represents a functionally relevant *in vivo* mechanism by which the more immunogenic DCs are efficiently selected. Indeed, following peripheral NK cell activation, DCs contained in the draining LNs were reduced in number but endowed with more potent T cell activating properties.

### The Editing of DCs by NK Cells Promotes Antigen Specific T Cell Expansion and a More Protective Immune Response During Cancer Cell Vaccination

**Figure 3:**
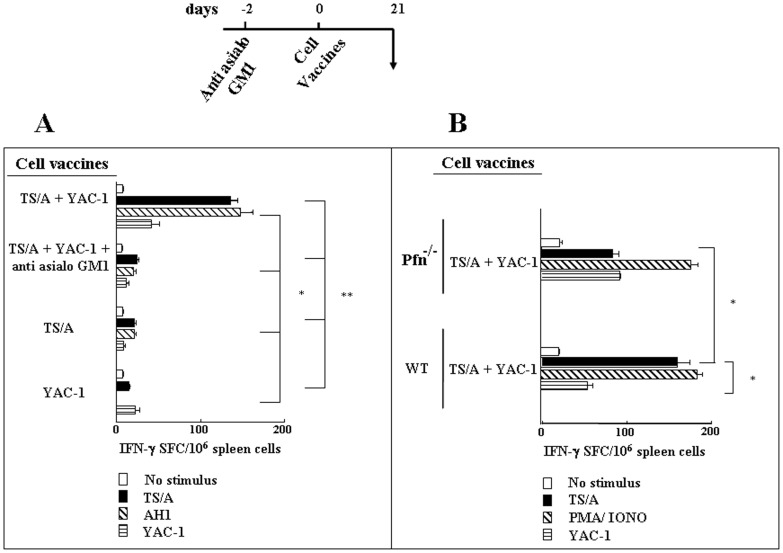
DC editing by NK cells promotes antigen specific CTL expansion. A: Mice were inoculated with immunogenic TS/A cells, TS/A cells mixed with MHC-devoid cells (YAC-1) or YAC-1 cells alone. In a group of mice, anti-asialo GM1 mAbs were administered i.p. 48 h before administration of cell vaccines to deplete NK cells. After 21 days, splenocytes were restimulated with either TS/A cells, the TS/A MHC class I-restricted immunodominant peptide AH1 or with YAC-1 cells and the frequencies of IFNγ-producing cells were determined by ELISPOT assay. A significant increase of antigen-specific IFNγ-producing cells was detectable in mice vaccinated with TS/A mixed with YAC-1 cells (TS/A + YAC-1). The increase in antigen-specific CTL was abrogated when mice had been depleted of NK cells before vaccination (TS/A + YAC-1 + anti asialo GM1). B: Similar experiments were performed in perforin KO mice (pfn ^−/−^) and in parallel with wild type (WT) mice. A significant lower number of TS/A tumor-specific CTL was induced by vaccination in perforin KO mice when compared to wild type mice. Bars represent mean values and SEM of results obtained in three independent experiments (three mice per group). ** =  p < 0,001; * =  p < 0,005.

Our data show that *in vivo* activation of NK cells results in a perforin-dependent decrease of LN dendritic cells, which is associated with the presence of more immunogenic DCs in draining LN. We therefore investigated whether it results in a more protective adaptive immune response in a cancer cell vaccination model. Groups of mice, which had either undergone NK cell depletion or mock treatment, were injected s.c. with the MHC-devoid cells (i.e. YAC-1 cells) and TS/A cells, a NK cell-resistant mammary adenocarcinoma tumor cell line. As controls, groups of mice were injected with either TS/A or YAC-1 cells alone. After three weeks, spleens were collected and cells were restimulated in culture with TS/A tumor cells or the AH1 peptide, which is an immunodominant MHC class I- restricted epitope of TS/A gp70env antigen [36], [37]. Splenocytes from mice that had been injected with TS/A plus YAC-1 cells showed significantly higher numbers of IFN-γ producing cells upon stimulation as compared to NK cell depleted mice or control mice (p<0.05 and <0.01) (Figure 3,A). These data indicate that lysis of DCs by activated NK cells supports the expansion of antigen-specific T lymphocytes *in vivo*. No increase in IFN-γ producing cells was observed when cultures were restimulated by YAC-1 cells, indicating that the increase in IFN-γ producing cells was not associated with NK cell activation. In addition, restimulation by either TS/A or their immunodominant MHC class I-restricted epitope AH1 induced a similar increase of IFN-γ producing splenocytes, indicating that they were mainly represented by MHC-class I-restricted cytotoxic T lymphocytes (CTL).

To elucidate whether NK cell help for CTL expansion was in fact associated to NK cell-mediated DC lysis, we set the same experiment using groups of pfn^−/−^ mice, which lack NK cell cytolytic activity. Spleen cells from pfn^−/−^ mice injected with TS/A plus the MHC-negative cells YAC-1 contained a significantly lower number of IFN-γ producing T cells when compared to wild type mice that were also injected with TS/A plus YAC-1 (Figure 3,B). These results suggest that NK cell-mediated, perforin-dependent, lysis of DCs has a role in the optimal generation of antigen-specific T cells. However, the present results also offer, at least in part, an alternative interpretation, i.e. that NK cells can directly stimulate DC maturation, most likely by releasing pro-inflammatory cytokines (e.g. TNF-α), as previously demonstrated [21], [35]. Indeed, in perforin-deficient mice, the activation of anti-tumor T cells was not as low as observed in NK cell-depleted mice using anti-asialo treatment (Figure 3,A). Thus, the enhanced antigen-specific T cell response, detectable upon NK cell-mediated DC editing could represent the outcome of both DC killing and cytokine-mediated DC activation.

**Figure 4:**
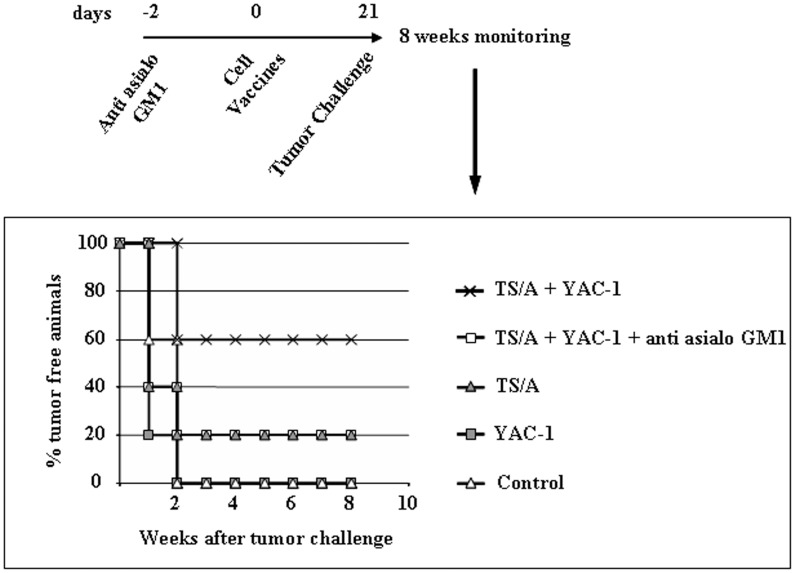
DC editing by NK cells generates a more protective immune response during cancer cell vaccination. Mice were vaccinated with immunogenic tumor cells alone (TS/A), tumor cells mixed with MHC-negative ells (TS/A + YAC-1) or MHC-negative cells alone (YAC-1) (five mice per group). In a group of mice, anti-asialo GM1 mAbs were administered i.p. 48 h before administration of cell vaccines to deplete NK cells. After 21 days, mice were challenged with a lethal dose of tumor cells and monitored for tumor growth twice a week for two months. Mice vaccinated with tumor cells mixed with MHC-negative cells (TS/A + YAC-1) displayed a delay in tumor growth and a striking survival to TS/A cancer cell challenge. This protective effect was abrogated when mice were depleted of NK cells (TS/A + YAC-1 + anti-asialo GM1).

Finally, we also observed that vaccination with irradiated TS/A cells plus YAC-1 cells resulted in a significant increase of survival of mice upon challenge with a lethal dose of TS/A cells (Figure 4). 60% of mice pre-injected with irradiated TS/A plus YAC-1 survived 8 weeks after tumor challenge whereas, in the same time interval, only 20% of either NK cell-depleted mice or mice pre-injected with irradiated TS/A cells alone survived the tumor challenge. Altogether, these results support the concept that the activation of NK cells induced by MHC-negative cells may promote a more protective immune response during cancer cell vaccination.

### Concluding Remarks

We have here shown that NK cells can elicit DC editing *in vivo*. NK cells, upon activation by MHC class I devoid cells, acquire the ability to select the most immunogenic myeloid DCs in draining LNs via a perforin-dependent mechanism. Although our current data do not constitute a formal proof of direct NK cell-mediated cytotoxicity against DCs, the abrogation of the phenomenon in pfn^−/−^ mice might indicate a direct DC lysis by NK cells.

Remarkably, this putative killing leads to the selection of more immunogenic DCs, characterized by a higher capacity to induce proliferation of allogeneic T cells. Thus, in addition to a direct stimulation of DCs mediated by NK cell-released cytokines, NK cells can contribute to T cell activation by selecting the most immunogenic DCs.

The identification of the type of NK cells responsible for the editing process, as well as the sites where DC killing allegedly occurs, still remains to be identified. In this context, it is possible that this event might take place in the periphery, where NK cells are equipped with a higher cytolytic activity as compared to those detected in lymph nodes. On the other hand, we cannot exclude that, under particular conditions (for example a cytokine storm), lymph node NK cells might switch their functional phenotype and acquire higher cytolytic potential, as previously proposed [38]. An alternative possibility is that peripheral NK cells displaying high cytolytic activity could migrate to lymph nodes following activation and acquisition of appropriate chemokine receptors, as suggested by recent reports [39]–[41].

The interaction between activated NK cells and DCs is also relevant for establishing a protective immune response. In a model of anti-cancer vaccination, administration of tumor cells together with NK activating MHC-devoid cells, boosted the expansion of tumor-specific CTLs resulting in enhanced survival of mice upon challenge with a lethal dose of tumor cells. Depletion of NK cells impaired this tumor-specific T cell response as well as its protective role against tumor challenge. Taken together, these results indicate that, *in vivo*, NK cells can edit DCs for the optimal generation of adaptive immune responses by an efficient selection of the more immunogenic DCs. Finally, our data also suggest that cancer cell vaccines could be improved by strategies aimed at NK cell activation, such as the use of NK-sensitive cancer cells.

## Materials and Methods

### Cell Lines, Animal Model and Experimental Conditions

The TS/A mammary adenocarcinoma murine cell line [36] (kindly provided by P.L. Lollini, University of Bologna, Bologna, Italy) was cultured in DMEM /10% FCS (Cambrex, Charles City, IA, USA). The YAC-1 (ATCC TIB-160) [42] was instead cultured in RPMI 1640/10% FCS (Cambrex, Charles City, IA, USA). 5–8 week-old female wild type BALB/c (H-2K^d^) mice and wild-type C57BL/6 (H-2^b^) mice were purchased from Harlan (Udine, Italy). Perforin^−/−^ mice (CByJ.B6-Prf1^tm1Sdz^/J) were purchased from The Jackson Laboratory (Bar Harbour, ME).

Mice were subcutaneously (s.c.) injected with 2 x 10^6^ irradiated (20,000 rads) YAC-1 cells or, where indicated, with either irradiated TS/A tumor cells alone (5 x 10^5^) or irradiated TS/A tumor cells (5 x 10^5^) admixed with irradiated YAC-1 cells (2 x 10^6^). Animals undergoing *in vivo* NK cell depletion received three intraperitoneal (i.p.) injections with anti-asialo-GM1 antibodies (anti-NK rabbit serum, 200 µl/mouse of 1∶10 diluted stock solution, Wako) at days −2/0/+1 as previously described [43] (day 0 was the day of cell vaccination. Tumor challenge was done at three weeks after vaccination with a tumorigenic dose 5 x 10^4^ of TS/A tumor cells. Tumor growth and size was measured twice weekly using a caliper. Injection of YAC-1 into BALB/c represents an allogenic system, but the absence of both MHC class I and class II expression in YAC-1 cells minimizes the potential alloresponse. The animals were housed in pathogen-free colony and experiments were performed according to the National Regulation on Animal Research Resources and approved by the Review Board of the Istituto Nazionale per la Ricerca sul Cancro, Genoa.

### Lymphoid Organ Cell Isolation

Inguinal lymph nodes (LNs) were surgically excised and collected in RPMI 1640/10% FCS. They were cut into small fragments using razor blades and digested in 2 mg/ml collagenase D and 30 µg/ml DNase I (Roche, Mannheim, Germany) at 37°C for 30 min. Single cell suspensions were prepared by filtration through a 70 µm cell strainer (BD Labware, San Jose, CA, USA). CD11c^+^ cells were isolated by positive selection using anti-CD11c microbeads and a magnetic separator (Miltenyi Biotec, Bergisch Gladbach, Germany). Spleen cells were isolated by mechanical dissociation and filtrate through a 70 µm cell strainer (BD Labware). Erythrocytes in the spleenic preparations were removed by hypotonic lysis with NH_4_Cl/ KHCO_3_ /EDTA.

### Antibodies and Flow Cytometry

Animals undergoing *in vivo* NK cell depletion received two i.p. injections with anti-asilao GM1 at day –2/0 (anti-NK rabbit serum, 200 µl/mouse of 1∶10 diluted stock solution, Wako Neuss, Germany). After 36 h from injection, inguinal LNs were harvested and the cell suspensions obtained were labeled with anti-CD11c, anti-DX5, anti-CD40, anti-CD80, anti-CD86 and anti-MHC class II (all from eBioscience, San Diego, CA ) and analyzed by flow cytometry (Facs Canto II, BD).

### Proliferation Assay

For the proliferation assay, spleen cells from B6 mice were labeled with 5 µM carboxyfluorescein succinimidyl ester (CFSE) in PBS plus 0.1% BSA for 10 min at 37°C. After extensive washing with PBS plus 0.1% BSA, cells were incubated with allogeneic CD11c DCs sorted from draining LN of mice injected with YAC-1 cells that had or had not been subjected to NK cell depletion. The DCs were extensively washed in PBS before culture with T cells. After 6 days, CFSE fluorescence was evaluated on CD3^+^ cells by flow cytometry.

### Enzyme-linked Immunospot Assays

The frequencies of IFN-γ-producing spleen cells from vaccinated animals were determined after three weeks by an enzyme-linked immunospot (ELISPOT) assay performed on splenocytes. Multiscreen-IP plates (Millipore, Bedford, MA and BD Pharmingen, San Jose, CA, USA) were coated overnight with 10 µg/ml of anti-IFN-γ mAb in PBS (endogen, Woburn, MA and BD Pharmingen, San Jose, CA, USA). Plates were then washed with RPMI 1640 and blocked for 3 h with PBS-2% bovine serum albumin. Splenocytes were counted in complete RPMI 1640 and then seeded at a 2-fold serial dilution, starting from 4×10^5^ cells per well, in duplicate, in the presence or absence of: (i) irradiated (20,000 rads) TS/A tumor cells; (ii) YAC-1 cells (both at 10∶1 effector/stimulator cell ratio); (iii) endogenous retroviral gp70-derived AH1 peptide, the 9-amino acid H-2L^d^-restricted peptide (SPSYVYHQF, synthesized by INBIOS S.r.l., Napoli, Italy) at final concentration of 10 µg/ml. AH1 is the immunodominant CD8 antigen expressed on surface of TS/A cancer cell lines [37]. Where indicated, cell stimulation was also obtained by PMA and ionomycin at final concentration of 50 ng/ml and 500 ng/ml, respectively (SIGMA). After 40 h of incubation, plates were washed with PBS-0.05% Tween 20 and incubated with 1 µg/ml biotinylated secondary mAb to IFN-γ (Endogen and BD Pharmingen, San Jose, CA, USA) in PBS-1% bovine serum albumin for 3 h at room temperature. Horseradish peroxidase-conjugated streptavidin (1∶5,000) was then added for 2 h at room temperature. After washing, the plates were stained with AEC staining kit (Sigma and BD Pharmingen, San Jose, CA, USA) and spots were counted using a stereomicroscope. A >2-fold increase in number of spots over the control (splenocytes cultured with no stimulus) was considered as a positive response. Data were expressed as number of spot-forming cells per million of spleen cells.

### Statistical Analysis

The statistical significance of differences was evaluated with Student’s t test or Mann Whitney test using Prism Graphpad software (GraphPad Software Inc., La Jolla, CA). All error bars represent SEM.
